# Economic evaluation of strategies against coronavirus: a systematic review

**DOI:** 10.1186/s13561-023-00430-1

**Published:** 2023-03-18

**Authors:** Reyhane Izadi, Nahid Hatam, Fatemeh Baberi, Setareh Yousefzadeh, Abdosaleh Jafari

**Affiliations:** 1 Department of Health Care Management, School of Management and Information Sciences, Shiraz University of Medical Sciences, Shiraz, Iran; 2 Health Human Resources Research Center, School of Management and Medical Informatics, Shiraz University of Medical Sciences, Shiraz, Iran; 3 Deputy of Research and Technology, School of Medicine, Shiraz University of Medical, Sciences, Shiraz, Iran; 4 Social Determinants of Health Research Center, Health Research Institute, Babol, University of Medical Sciences, Babol, Iran; 5 Health Human Resources Research Centre, School of Health Management and Information Sciences, Shiraz University of Medical Sciences, Shiraz, Iran

**Keywords:** Economic evaluation, Cost analysis, Strategy, COVID-19, Iran

## Abstract

**Background:**

The COVID-19 outbreak was defined as a pandemic on 11 March 2020 by the World Health Organization. After that, COVID-19 has enormously influenced health systems around the world, and it has claimed more than 4.2 million deaths until July 2021. The pandemic has led to global health, social and economic costs. This situation has prompted a crucial search for beneficial interventions and treatments, but little is known about their monetary value. This study is aimed at systematically reviewing the articles conducted on the economic evaluation of preventive, control and treatment strategies against COVID-19.

**Material and method:**

We searched PubMed, Web of Science, Scopus, and Google Scholar from December 2019 to October 2021 to find applicable literature to the economic evaluation of strategies against COVID-19. Two researchers screened potentially eligible titles and abstracts. The Consolidated Health Economic Evaluation Reporting Standards (CHEERS) checklist was used to quality assessment of studies.

**Results:**

Thirty-six studies were included in this review, and the average CHEERS score was 72. Cost-effectiveness analysis was the most common type of economic evaluation, used in 21 studies. And the quality-adjusted life year (QALY) was the main outcome applied to measure the effectiveness of interventions, which was used in 19 studies. In addition, articles were reported a wide range of incremental cost-effectiveness ratio (ICER), and the lowest cost per QALY ($321.14) was related to the use of vaccines.

**Conclusion:**

Based on the results of this systematic review, it seems that all strategies are likely to be more cost-effective against COVID-19 than no intervention and vaccination was the most cost-effective strategy. This research provides insight for decision makers in choosing optimal interventions against the next waves of the current pandemic and possible future pandemics.

## Introduction

The Coronavirus disease was diagnosed in December 2019, and the World Health Organization (WHO) defined its outbreak as a pandemic on March 2020. Since then, COVID-19 has profoundly affected health systems worldwide, causing more than 4.2 million deaths by the end of July 2021 [1]. The pandemic has led to global health, social and economic crises [2].

The total cost of the coronavirus and its pandemic was estimated to be roughly equivalent to 90% of the annual GDP (gross domestic product) in the United States [3]. A one-month lockdown in Tokyo led to an 86.1% (or 1.25 trillion yen) drop in daily production in Japan [4]. The average direct medical cost of an asymptomatic COVID-19 patient was $3,045 during the infection in the United States [5], and the global costs of the disease have been estimated from $77 billion to $2.7 trillion [6].

In Korea, the total Disability Adjusted Life Years (DALYs) for Coronavirus were estimated at 2,531 and 4.930 DALYs per 100,000 population; the Years Lost due to Disability (YLDs) and the Years of Life Lost (YLLs) constituted 10.3% and 89.7% of the DALYs, respectively [7]. Available studies have found that the highest total number of YLLs attributable to COVID-19 was in the United States, and the highest number of YLLs and DALYs per 100,000 people was in Belgium [8]. A study of five countries (United States (US), United Kingdom (UK), Canada, Norway, and Israel) found that the per capita value of YLL in US and UK was the highest and almost equal [9].

In order to prevent and control COVID-19, various non-pharmacological interventions have been implemented in different countries. Although these strategies can potentially lead to a significant reduction in productivity, the necessity of using them is unavoidable [2]. To date, treatment strategies to deal with corona disease are mainly supportive, such as oxygen supplementation and mechanical ventilation; unfortunately, there are no proven effective drugs against the coronavirus, and many drugs are used without conclusive evidence of their effectiveness and safety [10]. For some time, different types of COVID-19 vaccines have been used worldwide. Vaccines appear to be the only short-term solution to combat the coronavirus pandemic and must be distributed quickly, evenly, and efficiently [11]. In general, several strategies have been used to deal with COVID-19, some of the most important of which are quarantine, travel restrictions, screening, wearing masks, vaccines and social distancing [12]. Governments' response in choosing and applying these strategies is based on multiple economic, cultural, political, and ethical reasons [13]. For example, although the effectiveness of lockdown and restriction measures has been proven, they should be used cautiously due to their effects on individual freedoms and domestic violence [14].

So far, many studies have been conducted in various fields related to COVID-19. Early studies were focused on the knowledge of this virus [15, 16]. Many other studies have also examined the complications and costs caused by this epidemic [3, 17]. Some studies have compared other strategies against COVID-19 [18, 19]. To compare the cost-effectiveness of these strategies, outcomes such as QALY, DALY, prevented COVID-19 cases, and net benefit are often considered. And the costs are usually extracted and analyzed from three perspectives, including the payer, the health system, and the social [20–23]. Comparing the cost and outcome of interventions to deal with COVID-19 shows that an optimal response strategy is a combination of all interventions, and the form of this combination is strongly dependent on the characteristics of the growth of the epidemic; usually, the most cost-effective strategies included the four interventions of household contact tracing, isolation, mass symptom screening, and quarantine [24]. Understanding the benefits and burdens of these interventions, both individually and in combination, is essential for policymakers, and they often use different methods of economic evaluation to achieve this understanding. There are many types of economic evaluation for COVID-19 policies and interventions that differ in their methods of quantifying results and their approaches to aggregation. In a general classification, economic evaluations are divided into seven categories including cost, comparative effectiveness, cost-consequence, cost-effectiveness, benefit-cost, developed cost-effectiveness, and distributional cost-effectiveness [25].

Although many decision-makers use some kind of mental model to evaluate the advantages and disadvantages of different policy options, a detailed economic evaluation formalizes the decision-making process and makes decision-making more systematic, comprehensive and transparent [25, 26]. On the other hand, policymakers need to know which strategy has a positive effect on the control and prevention of COVID-19, or which types of medical and non-medical interventions are more necessary to prevent this disease [27]. It seems that a comprehensive review of economic evaluation studies of solutions to deal with COVID-19 can clarify the path of optimal allocation of resources for decision-makers. Therefore, this research was conducted to summarize the cost-effectiveness of strategies to deal with COVID-19.

## Method

### Search methods for identification of studies

This systematic review followed the PRISMA (Preferred Reporting Items for Systematic reviews and Meta-Analyses) flow diagram [28]. This study was conducted to review published studies on the cost-effectiveness of strategies against COVID-19 from December 2019 to October 2021. We developed a search strategy to identify studies using the PICOS (Population/Problem-Intervention-Comparison-Outcome- Study Design) framework. The studies were extracted from the following databases: PubMed, Scopus, Web of Science, and Google Scholar. Keywords based on MeSH (Medical Subject Headings) terms were placed into two categories ("COVID-19" and "cost"). The logical operator "OR" was used between all synonymous keywords, and then the first and second-category keywords were merged with the logical operator "AND". Endnote X7.1 software was used to manage references. The selected studies focused on the economic evaluation of the response programs for COVID-19.

### Selection of studies

We identified potentially eligible titles and abstracts based on inclusion/exclusion criteria. Then, two authors independently evaluated the text of the selected articles. Disagreements concerning including eligible studies were resolved by a third author. The inclusion and exclusion criteria are listed in Table 1.

**Table 1:** Eligibility criteria

	**Inclusion criteria**	**Exclusion criteria**
Population	General population or targeted population	NA
Intervention	All strategies to deal with COVID-19 (preventive, control, or treatment)	Other interventions
Comparator	Any other intervention, no intervention	NA
Outcome	Incremental cost per QALY, Incremental cost per DALY, Cost per case averted, Cost per death averted,	Cost analysis studies (i.e., studies which measured or compared costs without health outcomes) or outcomes related to effectiveness only
Cost perspective	No restriction	NA
Study design	Full economic evaluation studies (CEA, CUA or CBA), Partial economic evaluation studies (if both costs and outcomes of an intervention were included)	Conference abstracts, review articles, animal studies and is do not find the full text
Context	No restrictions (all countries)	NA
Language	English language	NA

*CEA* cost-effectiveness analysis, *CUA* cost-utility analysis, *CBA* cost–benefit analysis, *QALY* quality-adjusted life years, *DALY* disability-adjusted life years, *NA* not applicable

### Data extraction and analysis

The CHEERS checklist (with 24 items) was used to report the quality of the studies [29]. The items in this checklist were evaluated and scored as “fully met = 1”, “not meet = 0”, “partially met = 0.5”, or “not applicable”. Then we determined the percentage of the score for each study. Articles were divided into four categories based on the percentage of points earned: poor quality (scoring < 55%), good quality (55–70%), very good quality (70–85%), excellent quality (scoring ≥ 85%); poor studies were excluded from the analysis.

The selected studies were fully reviewed, and the required data were extracted and summarized. We designed a data extraction form that included the following information: study population, country, compared interventions, time horizon, perspective, type of economic evaluation, outcomes, and costs.

In order to analyze and compare the results of different studies, we first converted all studies into the same currency (US dollar). Given that the studies were conducted in different years (2019 to 2021), we updated the results of the studies to 2021 according to the inflation rate of the countries. And finally, we compared the studies with the same outcomes.

## Results

### Review profile

**Fig. 1:**
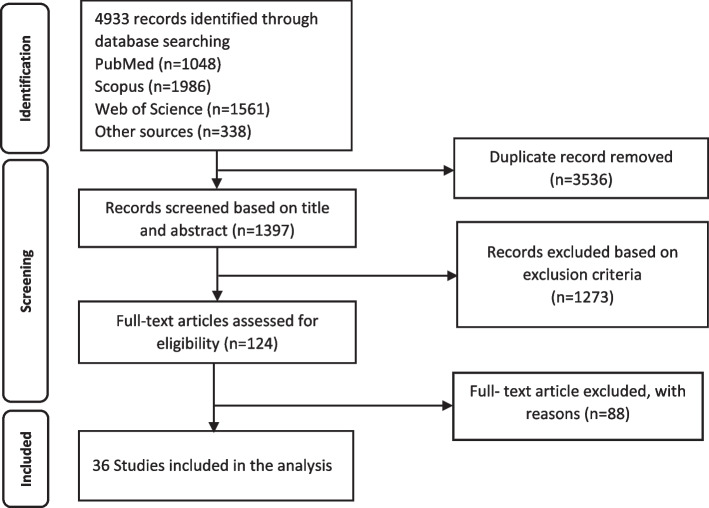
PRISMA flow chart for study selection

The PRISMA diagram is shown in Fig. 1. The search in all electronic databases identified a total of 4933 records. After removing duplicate records, 1397 articles were eligible based on screening the titles and abstracts. After that, 124 records met the inclusion criteria and were selected for full-text evaluation. Finally, 36 studies were considered in our analysis, which has been presented in Table 2.

**Table 2:** Study design and setting overview

Category	Author	Country/ Year	population	Alternative options for comparison	Type of economic evaluation	Study perspective	Outcome measure	Time horizon	Included Cost	Discount rate	Sensitivity analysis	CHEERS	ICER^a^/ NMB^b^/ CBR^c^
S1, S2, S3	Shlomai A, et al. [30]	Israel/ 2020	General population	1. National lockdown 2. Testing, tracing and isolation	CEA (SEIR^d^ model)	NR^e^	Death averted	200 days	Direct medical cost (cost of infected individuals = isolation, hospitalization, ICU)	0% (NA^f^)	One -way	0.91	The ICER value per death prevented was $45,104,156 (*45,776,207.92 *^*t*^), equivalent to $4.5 *(*^*t*^*$4.56)* million per QALY), in national lockdown )The ICER threshold value was estimated at around $15,243–17,366 per QALY)
S8	Zhao J, et al. [31]	China/ 2019	General population	Implementation of MRP^g^: 1. No delay 2. 1-week delay, 3. 2-week delay, 4. 4-week delay,	CUA	Societal	DALY	NR	Direct and indirect cost (average cost of hospital stay, weighted quarantine cost for all suspected cases, productivity loss)	3%	One-way and PSA^h^	0.81	NMB (billion/$) for strategies1, 2, 3, and 4 was − 381 *(-394.04*^*t*^*)*, − 658 *(-680.52*^*t*^*)*, − 910 *(-941.15*^*t*^*)*, − 3,285 *(-3,397.46*^*t*^*)*, respectively (Willingness-to-pay RMB^i^ 70,892 per averted DALY)
S7	Hagens A, et al. [32]	Turkey/ 2019	General population	1. Without vaccination 2. Vaccination (scenario1, equal effectiveness on transmission and disease (90% effectiveness) / scenario2, limited effectiveness on transmission (90% on disease and 45% on transmission))	CEA	Healthcare system	QALY	1 year	Direct and indirect cost (hospitalization costs, ICU stay, pharmacotherapy at home, vaccination, decreased productivity)	3%	Multi-way	0.85	ICER = 511 *(686.20*^*t*^*)* $/QALY (for equal effectiveness on transmission) and 1,045 *(1,403.29*^*t*^*)* $/QALY (for limited effectiveness on transmission)
S2, S5, S8	Wang Q, et al. [33]	China/ 2021	General population	1. Single strategy (personal protection, isolation-and-quarantine, community containment) 2. Joint strategy (personal protection and isolation-and-quarantine, personal protection and community containment^j^) 3. No-intervention	CEA	Societal	Number of cases avoided	14 days	Direct and indirect cost (surgical mask, soap, water cost, direct medical cost per case, lost disposable income, quarantine cost)	NA	One-and-two-way	0.77	ICER value compared to no intervention: Isolation-and-quarantine was still the most cost-effective single strategy (ICER = $1,278.43) The joint strategy of personal protection and isolation-and-quarantine was the optimal choice ($1*,*000 per case avoided) )ICER threshold value = $ 9,595(
S7	Wang W–C, et al. [34]	Taiwan (China) / 2021	General population	1.1.1.1.Moderna vaccination 2.2.2.2.Pfizer vaccination 3.3.3.3.AstraZeneca vaccination 4.4.4.4.No vaccination	CUA (Markov model)	Societal	QALY,	180 days	Direct medical cost and indirect cost (vaccine, treatment, productivity loss)	NA	One-way	0.58	ICUR^k^ per QALY ($): Pfizer = − 356.75 AstraZeneca = − 341.43 Moderna = − 321.14 )Willingness-to-pay threshold = $50,000(
S4, S6, S7	Padula WV, et al. [35]	United States/ 2020	General population	1.Do Nothing 2.Social distancing, 3.COVID-19 treatment 4. COVID-19 vaccine	CEA (Markov model)	Healthcare system	QALY	1 year	Direct medical cost and indirect cost (cost of a lost work-day, vaccine, ICU bed, hospital bed, emergency care visit (tests and x-ray), urgent care visit, (tests and x-ray), primary care visit (tests and x-ray))	3%	PSA	0.62	Budget impact of the vaccination was $40 *(41.88*^*t*^*)* per person, making it the more affordable option (vs. $102 *(106.79*^*t*^*)*, for do nothing) ICER ($/QALY): $16/ 0.02 = $800 *(837.60*^*t*^*)* per QALY )Willingness-to-pay threshold = $100,000/QALY(
S7	Kohli M,et al. [36]	United States/ 2020	General population (People over 18 years old)	1. Vaccination 2. No vaccination	CEA (Markov model)	Healthcare system	QALY	1 year	Direct medical cost (vaccine, ambulatory care only, hospitalization without ICU or ventilator, hospitalization with ICU, hospitalization with ICU + ventilator)	3%	Base case scenario and tornado diagram	0.75	ICER = $8,200 *(8,585.40*^*t*^*)* per QALY )Willingness-to-pay threshold = ranged from $50,000 to $150,000 per QALY gained(
S1	Jiang Y, et al. [37]	China/ 2020	COVID‐19 patients	Three versus two reverse transcription-PCR (RT-PCR) tests for diagnosing and discharging people with COVID-19	CEA (SIR^l^ model)	Healthcare system	QALY	43 days	Direct medical cost (cost of RT-PCR test, cost per hospital day of the fully quarantined individuals)	5%	One-way	0.66	Net monetary benefit (as replacement for ICER) = CN¥104 million *($16.52 million*^*u*^*)* in 43 days
S2	Melia A, et al. [38]	Australia/ 2020	General population in 3 categories (1. Juniors 2.Adult 3.Seniors)	1. Home isolation 2. Hotel room isolation	CEA (Decision tree)	Government	Secondary household infection rate	14 days	Direct cost (ward cost, ICU cost, isolation cost at the hotel)	NA	NR	0.58	Hotel isolation vs. home isolation, in NSW^m^ = AU$3*,*000 *(2,243.37*^*u*^*)* per person vs. AU$1054 *(788.16*^*u*^*)* per person (NMB = AU$1*,*946 *(1,455.19*^*u*^*)* per person) Hotel isolation vs. home isolation, in WA^n^ = AU$2,520 *(1,884.43*^*u*^*)* per person vs. 955.5 *(714.15*^*u*^*)* per person (NMB = AU$1*,*564.5 *(1169.91*^*u*^*)* per person)
S5	Risko N, et al. [39]	low- and middle-income countries/ 2020	Health workers	1. Adequate of personal protective equipment 2.Inadequate of personal protective equipment	CEA	Societal	HCW^o^ cases averted, HCW death averted,	30 weeks	Direct and indirect cost (training costs, costs of labor and healthcare utilization, lost future productivity due to early mortality)	NR	Bayesian multivariate sensitivity analysis	0.79	ICER = $59 *(61.01*^*t*^*)* per HCW case averted, ICER = $4,309 *(4,456.36*^*t*^*)* per HCW life saved,
S4	Thunström L, et al. [40]	United States/ 2020	General population	1. With Social distancing 2. Without social distancing	CBA	NR	VSL	30 years	Indirect cost (lost GDP^p^)	3%	Break even sensitivity analysis	0.83	NMB = $5.2 *(5.44*^*t*^*)* trillion
S1, S2	González Lopez-Valcarcel B, et al. [41]	Spain/ 2020	High-risk individuals	1.Test-tracking quarantine (TTQ) strategy 2. Do nothing	CBA	Societal	QALY	2 years	Direct and indirect cost (tests, tracers, COVID-19 cases treated at home, hospitalization (ICU, non-ICU), cost due to premature mortality and long-term morbidity consequences, lost GDP due to COVID-19 outbreak and outbreak responses)	3%	Base case sensitivity analysis	0.91	Benefit-to-cost ratio (excluding health and morbidity) = €7 *(8.20*^*u*^*)* savings for every euro spent on TTQ Benefit-to-cost ratio (including health and morbidity) = €19 *(22.26*^*u*^*)* savings for every euro *(1.17*^*u*^*)* spent on TTQ
S1, S4, S5	Losina E,et al. [42]	United States/ 2020	Undergraduate students and faculty at colleges	1.Social distancing + masks 2. Masks alone 3. laboratory screening	CEA	Societal	QALY, Number of infections prevented	105 days	Direct cost (cost of isolation, testing and hospitalization, and NPIs (costs of running and maintaining online education platforms, masks and cleaning and disinfecting measures))	NA	Base case sensitivity analysis	0.66	ICER = $170 *(177.99*^*t*^*)* per infection prevented, ICER = $49*,*200 *(51,512.4*^*t*^*)* per QALY saved,
S5	Bagepally BS, et al^.^ [43]	India/ 2019	General population	1.Surgical masks, 2.N-95 respirator (non-fit tested) 3.N-95 respirator (fit tested) 4.Hand hygiene 5.Surgical mask with hand hygiene 6. No intervention	CUA (Decision tree and Markov model)	Healthcare system	QALY	1 year	Direct cost (cost of quarantine, isolation, paracetamol, outpatient, cost of disease (mild, severe, critical), cost of surgical mask, cost of N95 respirator, cost of hand hygiene, cost of COVID-19 test)	NA	One-way and PSA	0.62	ICER (million $/QALY): Surgical with hand hygiene = $1.12*(1.25*^*t*^*)* Hand-hygiene alone = $0.113*(0.12*^*t*^*),* Surgical-mask alone = $1.03*(1.15*^*t*^*),* N- 95 respirator, fit tested = $5.65*(6.33*^*t*^*),* N- 95 respirator, non-fit tested = $2.98*(3.34*^*t*^*),* (Cost-effectiveness threshold = $1*,*921)
S6	Sheinson D, et al. [44]	United States/ 2020	Hospitalized patients with COVID-19 & 62.5 + years old	1. No oxygen support 2. Oxygen support with ventilation 3.Oxygen support without ventilation	CEA (Markov model)	Societal	QALY	Lifetime	Direct and indirect cost (annual healthcare costs after discharge, productivity losses, inpatient costs (mechanical ventilation, oxygen support without ventilation, no oxygen support, mechanical ventilation bundled payment, oxygen support without ventilation bundled payment, no oxygen support bundled payment), drug costs)	3%	One-way and PSA	0.77	ICER ($/QALY) = $8*,*028 *(8,508.70*^*t*^*)* per QALY
S1	Paltiel AD, et al. [45]	United States/ 2020	General population	1.Home-based SARS-CoV-2 antigen testing, 2. No test intervention	CEA	Societal	Infection averted, Death averted,	60 days	Direct medical cost and indirect cost (cos of tests, inpatient care, and lost workdays)	NA	NR	0.62	ICER = $7*,*890 *(8,260.83*^*t*^*)* per infection averted, ICER = $1*,*430*,*000 *(1,497,210*^*t*^*)* per death averted,
S1	Abdalhamid B, et al. [46]	United States/ 2020	Asymptomatic and symptomatic patients with COVID-19	1.RNA extraction & RT-PCR in pool testing 2.Individual testing	CEA	NR	Number of diagnosed patients	NR	Direct medical cost and indirect cost (reagents and consumables, labor)	NR	NR	0.58	ICER = $35*,*134 *(36,785.30*^*t*^*)* per each diagnosed case
S1	Neilan AM, et al. [47]	United States/ 2020	General population	1. PCR for people with symptoms + no symptoms, 2. PCR only for symptoms,	CEA (Microsimulation model)	Health care system	QALY	180 days	Direct medical cost (SARS-CoV-2 PCR assay, hospital bed, ICU)	For cost: NA, For life years lost:3%,	Multi-way and one-way	0.79	ICER = $33*,*000 *(34,551*^*t*^*)* /QALY for Symptomatic + asymptomatic monthly (Willingness-to-pay threshold = $100*,*000/QALY)
S1, S2, S4, S5	Asamoah JKK, et al. [48]	Ghana/ 2020	General population	1. The effective testing and quarantine when boarders are opened 2.Intensifying the usage of nose masks and face shields through education 3. Cleaning of surfaces with home-based detergents 4.Safety measures adopted by the asymptomatic and symptomatic individuals^q^ 5.Fumigating commercial areas such as markets	CEA (Deterministic model)	NR	Prevented infection cases	56 days	Direct and indirect cost (intervention costs, averted disease costs and costs of prevented cases)	NA	One-way	0.58	ICER = $7.1362^t^10^−12^ per infection averted *(7.8476*^*t*^*10 *^*−12t*^*)*
S3	Ryan AJAaS, et al. [49]	Ireland/ 2019	People unemployed	Lockdown in 2 scenarios: 1. Generous, 2. Conservative,	CBA	Government	QALY	3 years	Direct and indirect costs (GDP deficit, government spending on healthcare)	3%	NR	0.70	Conservative policy: The total cost per QALY is €28,000 *(32,489.78*^*u*^*),* €2 billion divided by 71,428 QALYs Generous policy: The total cost per QALY is €15,555 *(18,049.23*^*u*^*)*, €1 billion divided by 64,285 QALYs) )The costs of lockdown are 25 times greater than its benefits(
S6	Águas R, et al. [50]	United Kingdom/ 2020	Hospitalized COVID‐19 patients needing oxygen and ventilation	1.Medication with dexamethasone, if patient has criteria for this treatment 2.No-Medication with dexamethasone	CEA	Health care provider	QALY	6 months	Direct medical cost (daily hospital patient costs per treatment)	NA	NR	0.68	ICER < £20,000 *(27,768.56*^*u*^*)* per quality-adjusted life-year (QALY) England has an explicit threshold range of £20,000–£30,000
S3	Rowthorn R, et al. [51]	United Kingdom/ 2020	General population	1.Do nothing 2.Lockdown	CBA (SIR model)	Governments	Deaths	1 week	Direct medical cost and indirect cost (treatment, loss of output(production))	NA	NR	0.70	ICER = £2 *(2.77*^*u*^*)* million to each fatality )A 10-week lockdown is only optimal if the value of life for COVID-19 victims exceeds £10 m.(
S1, S2	Paltiel AD, et al. [52]	United States/ 2020	College students	1.Screening (every 2 days) and isolation program, 2.Screening (daily) and isolation program, 3.Screening (weekly) and isolation program,	CEA	Societal	Infectious case prevented	80 days	Direct medical cost (equipment and personnel costs)	NA	NR	0.58	ICER = $7900 *(8271.30*^*t*^*)* per infected averted, (Willingness-to-pay = $100,000(
S3, S8	Broughel J, et al. [53]	United States/ 2020	General population	1.No intervention 2. State suppression policies (stay-at-home; closing nonessential businesses, public schools, higher educational and facilities; severe travel restrictions)	CBA	Societal	QALY	NR	Direct and indirect cost (lost income, costs of suppression measures, hospitalization, ICU admission, mechanical ventilation)	5%	NR	0.58	Gross mortality benefits using the “value-of-production” approach: Net benefit, low = $285.3 billion *(298.70*^*t*^*)* Net benefit, high = $368.3 billion *(385.61*^*t*^*)* (The monetary threshold per QALY (gross mortality benefits): between $285 billion and $530 billion)
S6	Gandjour AJm,et al. [54]	Germany/ 2020	COVID‐19 patients	1.No intervention 2.Provision of additional capacity (ICU bed)	CEA	Societal	Life year gained	Lifetime	Direct medical cost (initial stay in ICU, rehospitalization)	3% for costs & 1% for health benefits	One‐way	0.70	ICER = €24,815 *(29,109.55*^*u*^*)* per life year gained (ICER of an additional ICU bed was €24,815 per life year gained) (The willingness to pay = €101,493 per life year gained)
S4	Schonberger RB, et al. [55]	United States/ 2020	General population	1.Strategy of full reopening aimed at achieving herd immunity 2.Strategy of limited reopening with social distancing	CBA	NR	QALY	8 months	Indirect cost (plausible effects of economic cost on US GDP)	3%	NR	0.58	ICER = $125,000 *(130,875*^*t*^*)* per QALY
S7	Sandmann, F.G [56]	United Kingdom/ 2020	General population (individuals aged 20 years or older)	1.Vaccination (best-case scenario, worst-case scenario), 2.No vaccination	CBA (A dynamic modelling framework)	Health system	QALY	10 years	Direct and indirect cost (hospital admissions (ICU, non-ICU), personal protective equipment, visits to general practitioners, remote helpline calls, adverse events following immunisation, vaccine administrations, vaccine costs, conservative long-run cost per vaccine dose)	3·5%	PSA	0.83	Incremental net monetary values ranging from £12 *(16.66*^*u*^*)* billion to £334·7*(464.70*^*u*^*)* billion in the best-case scenario; And, from –£1·1 *(1.52*^*u*^*)* billion to £56·9 *(79.00*^*u*^*)* billion in the worst-case scenario (Monetary value threshold per QALY = £20,000)
S3	Dutta M, Husain Z [57]	India/ 2020	General population	1.lockdown under 3 alternative scenarios (growth in income: 6%,7%, and 8%)	CBA	Health system	Cases avoided, Deaths averted	14 days	Direct and indirect cost (homecare, hospitalization, ICU admission, unemployment, loss in production)	4%	NR	0.70	Under all the scenarios: Net benefits < 0
S5	Kazungu J,et al. [58]	Kenya/ 2020	Healthcare workers	1. Adequate/full PPE utilization 2. Inadequate supply of PPE	CEA	Government	Death averted, COVID-19 case averted,	1 year	Direct and indirect cost (cost of training (nurse, clinical officer), cost per nurse-day of work, hospital bed, lost GDP per capita)	NA	PSA	0.72	Cost per COVID-19 case averted = $51 *(54.11*^*t*^*)* Cost per death averted = $ 371 *(393.66*^*t*^*)* (Willingness to pay = $517)
S6	Chow R,et al. [59]	United States/ 2020	Hospitalized COVID-19 patients	1.Statin use 2.No statin use	CEA	Healthcare system	Discharged; Death; Toxicity	4 weeks	Direct medical cost (hospitalization (ICU, non-ICU), Statin)	NA	NR	0.83	ICER < 0 The mean cost for patients receiving statins was $31,623 *(33,109.28*^*t*^*)*, whereas the mean cost for patients not receiving statins was $33,218*(34,779.24*^*t*^*)*, The mean effectiveness for the two cohorts were 1.73 and 1.71, respectively
S6	Jo Y,et al. [60]	South Africa/ 2020	Hospitalized COVID-19 patients in ICU	1.Administration of dexamethasone to ventilated patients and Remdesivir to non-ventilated patients, 2.Dexamethasone alone to both non-ventilated and ventilated patients, 3.Remdesivir to no ventilated patients only, 4.Dexamethasone to ventilated patients only 5. All relative to a scenario of standard care	CEA	Healthcare system	Deaths averted	6 months	Direct medical cost (cost of Remdesivir regimen, cost of dexamethasone regimen, ICU)	5%	One-way and three-way sensitivity analyses	0.87	ICER = $231 *(241.64*^*t*^*)* per death averted
S6	Jiang Y,et al. [61]	China/ 2020	Severe COVID-19 patients	1.Remdesivir regimen 2.Standard of care	CEA	Healthcare system	QALY	3 months	Direct medical cost (RT-PCR test fee for diagnosis and discharge of all infected and symptomatic persons, 1-time outpatient costs of mild patients, bed costs of mild patients during quarantine, hospitalization costs of moderate patients, hospitalization costs of severe patients, SoC^r^ medication costs of moderate patients, SoC medication costs of severe patients, and Remdesivir acquisition costs)	5%	One-way and PSA	0.81	ICER = CN¥ 14,098 *(2,239.86*^*u*^*)* per QALY
S6	Congly SE, et al. [62]	United States/ 2020	COVID-19 patients	1.Remdesivir to all patients, 2.Remdesivir in only moderate and only severe infections, 3.Dexamethasone to all patients, 4.Dexamethasone in severe infections, 5.Remdesivir in moderate/dexamethasone in severe infections,	CUA	Payer	QALY	1 year	Direct medical cost (supportive care)	NA	PSA	0.87	ICER = $980.84 *(1026.93*^t^*)* per QALY (Willingness to pay threshold = $100,000/QALY)
S2, S3, S4, S5, S8	Lally M [63]	New Zealand/ 2021	General population	1.Lockdown strategy 2. Mitigation strategy (including case isolation, quarantining of members of their households, limiting large gatherings, social distancing, the wearing of masks on public transport, and restrictions targeted at only high-risk groups)	CBA	NR	QALY	4 months	Indirect cost (GDP Losses)	3.5%	NR	0.64	lockdown: Cost per QALY saved = at least $924,000 *(960,405.60*^t^*)* (A threshold figure of $62,000 for health interventions in New Zealand)
S1, S2, S3, S6	Raitzer D,et al. [64]	Philippines/ 2020	Students	1.Increased tracing- testing-and isolation TTQ 2.Paid sick leave (in cases with a positive diagnosis for treatment) 3.School face-to-face closure (in 3 scenarios: for all; for 15 + year old; for under 15-year old)	CBA	NR	Reduced disease burden of covid-19, Reduced cost of treatment, Life saved	Lifetime	Direct and indirect cost (labor force to care for children at home, lost income, lost productivity caused by poor training, employment of private school teachers)	Less than 3%	NR	0.70	Benefit–cost ratio (policy1:1.20, policy2: 10, policy3: 0.011) (₱768 *(15.65*^*u*^*)* million per life saved for closure at all levels, ₱366 *(7.45*^*u*^*)* million per life saved from closure for 15 + year old, and ₱1.38 *(0.02*^*u*^*)* billion per life saved from closure for those under 15 years of age) (Willingness to pay = ₱10,000)
S1	Seguí FL,et al. [65]	Spain/ 2020	General population	Mass COVID-19 screenings of an asymptomatic population (Scenario1: PCR test & scenario2: RAT^s^)	CBA	Societal	Monetary value of a QALY	5 months	Direct medical cost (hospitalization, ICU admission)	3%	NR	0.75	Benefit–cost ratio: Base case = 1.20, RAT = 1.63; PCR = 1.23 (Monetary value of a QALY: €25,000)

^a^Incremental Cost-Effectiveness Ratio

^b^Net Monetary Benefit

^c^Benefit-Cost Ratio

^d^Susceptible Exposed-Infected-Recovered (SEIR)

^e^NR: Not Reported

^f^NA: Not Applicable

^g^Movement restriction policies

^h^Probabilistic Sensitivity Analysis

^i^Renminbi

^j^In this study, community containment was a restriction on the movement of people within a community

^k^Incremental Cost-Utility Ratio

^l^Susceptible-Infected-Recovered (SIR)

^m^New South Wale

^n^Western Australia

^o^Health care worker

^p^gross domestic product

^q^Such as practicing proper coughing etiquette by maintaining a distance, covering coughs and sneezes with disposable tissues or clothing and washing of hands after coughing or sneezing

^r^Standard of care

^s^Rapid antigen test (RAT)

^t^Adjusted for inflation rate until 2021

^u^Adjusted in terms of US dollars and inflation rate until 2021

### Study characteristics

The characteristics of the studies are summarized as follows: country setting and year of study, target population of the study, alternatives for comparison, type of economic evaluation used for data analysis, outcome measure for effectiveness, time horizon, perspective of the study, included cost, type of sensitivity analysis, the discount rate for costs and outcomes, and the incremental cost-effectiveness ratio/net monetary benefit (Table 2).

In total, 36 studies were included in this review. The average score of CHEERS was 72. Five articles with excellent quality (85 or higher), seventeen articles with good quality (70 to 85), and fourteen articles with average quality (55 to 70) were included in this review. Most of these studies were from the United States (*n* = 13) and China (*n* = 5). In more than half of the articles (*n* = 20), the target population of the study was the general population. In terms of the type of economic evaluation, most studies used cost-effectiveness analysis (*n* = 21). Except for seven articles, all of them had a study perspective. The findings of this review study show that 20 studies (55.55%) used the discount rate and in 14 studies (38.88%), as the period of the study was limited, the discount rate was not used; two studies did not report this. All studies clearly stated the time horizon of the study, except for three studies. The time horizon of most studies (*n* = 26) was one year or less.

The most common outcome used to measure the effectiveness of interventions was QALY, which was used in 19 studies (52.77%). One-way sensitivity analysis was the most common type of sensitivity analysis in the reviewed studies and was used in 12 studies (33.33%), the probabilistic sensitivity analysis was used in four studies (11.11%), and in four studies, both have been done simultaneously. Sensitivity analysis is used to investigate the effect of uncertainty in the results and the generalizability of the results [66]. The findings of this review also indicated that the studies included a wide range of direct (medical and non-medical) and indirect (especially lost production) costs. Thirteen articles (36.11%) generally studied direct and indirect costs. In 17 articles (47.22%), direct medical costs have been included, and in four of these articles, indirect costs have also been investigated simultaneously. Furthermore, the articles reported a wide range of incremental cost-effectiveness ratios. The highest and lowest costs per QALY were observed in India ($6.33 million for N-95 respirator) and Taiwan ($321.14 for the Moderna Vaccine), respectively. It was also found that China ($-3,397.46 billion to implement movement restriction policies with a delay of four weeks) and the United States ($5.44 trillion for the implementation of social distancing) had the lowest and highest net monetary benefit, respectively.

### Summary of the economic evaluation of strategies against COVID-19

The reviewed economic evaluation studies were different vastly based on type of interventions, setting, perspectives, used methods, and populations. For this reason, direct comparing the results of studies was difficult. Complete information on these studies is provided in Table 2. In this section, the most important information of an economic evaluation study is presented separately for each study (this information includes: economic evaluation method, alternatives, country, and the result of the study in US dollars based on ICER / NMB (Net Monetary Benefit) / CBR (Benefit–Cost Ratio).

The results showed that the strategies to deal with COVID-19 were included in eight general categories. The three common strategies in the studies were screening and diagnostic tests (n_S1_ = 11), quarantine and isolation (n_S2_ = 8), and therapeutic interventions (n_S6_ = 9). A handful of articles evaluated social distancing (n_S4_ = 6), personal protective equipment (n_S5_ = 7), lockdowns (n_S3_ = 7), vaccination (n_S7_ = 5), and travel restriction (n_S8_ = 4).

#### Screening and diagnostic tests strategy (S1)

In 11 articles, the strategy of "screening and diagnostic tests" has been studied in combination with other interventions or individually. Although there was limited evidence that this strategy was less cost-effective than social distancing with masks [67], paid sick leave for treatment [68], and cleaning surfaces as a protective measure [69], in eight studies, different forms of this strategy were clearly introduced as the dominant and cost-effective option compared to the competing option (other strategies, non-intervention, or another form of this strategy).

Four studies in the United States studied different forms of screening and diagnostic tests strategy using the CEA method. In one of these studies, home-based SARS-CoV-2 antigen testing was found to be superior to no intervention; the ICER per death prevented and infection prevented were $1,497,210 and $8,260.83, respectively [23]. Also, RT-PCR (Reverse Transcription Polymerase Chain Reaction) pool tests were the dominant option compared to individual tests with an ICER of $36,785.30 per diagnosed case [70]. Additionally, PCR for all was found to be superior compared to PCR for symptomatic individuals, with an ICER of $34,551 per QALY [71]. It was also found that the combined strategy of screening (every other day) with isolation is a more cost-effective option compared to daily and weekly screenings and saves $8,271.30 per infection prevented [72]. A CBA study from Spain showed that the use of diagnostic tests in the combined TTQ (Test-Tracking-Quarantine) strategy is the dominant option compared to no intervention, and the benefit–cost ratio for it was $22.26 [73]. And another study from this country with the same method showed that for mass screening of asymptomatic population for COVID-19, rapid antigen test was superior to PCR test with a benefit–cost ratio of 1.63 versus 1.20 [74]. A CEA study in China found that performing three RT-PCR tests compared to two tests was the superior option for diagnosing and discharging people with COVID-19, with a net monetary benefit of $16.52 million in 43 days [75]. A study from Israel using the CEA method showed that national lockdown was inferior to an alternative combined strategy of testing, tracing, and isolation; regarding national lockdown, the ICER was $45,776,207.92 per death averted, which was higher than the willingness-to-pay threshold [20].

#### Quarantine and isolation strategy (S2)

Eight studies investigated the "quarantine and isolation" strategy in combination with other interventions or separately. Although this strategy was mentioned as a less cost-effective option than paid sick leave for treatment [68] and cleaning surfaces [69], different forms of this strategy were superior to other alternatives in 6 articles.

A study from China using CEA showed that the isolation-and-quarantine strategy was superior to the personal protection and community containment strategies (ICER per case averted = $1,278.43). The joint strategy of personal protection and isolation-and-quarantine was also introduced as a more cost-effective option than personal protection and community containment (ICER per case avoided = $1,000) [76]. Another study using the same method in Australia found that home isolation was a more cost-effective option than hotel isolation, and the net monetary benefit per person was estimated at $1,455.19 in New South Wales and $1,169.91 in Western Australia [77]. Also, a CBA article from New Zealand showed that the combined mitigation strategy, which included isolation and quarantine, was a more cost-effective option than the lockdown strategy; with the implementation of the lockdown strategy, the cost per QALY was $960,405.60, which was much higher than the willingness-to-pay threshold [78]. In three other studies, which were also mentioned in the previous section, the combined strategies of screening (every other day) with isolation [72] and TTQ [20, 73] were dominant compared to other alternatives.

#### Lockdown strategy (S3)

Lockdown strategy was investigated in seven studies in combination with other interventions or individually. For analysis, the CBA method was used in six studies and CEA method was used in one. In these studies, different forms of this strategy were compared with other strategies or no intervention, and the results in one study showed that this strategy was the dominant option.

One study from the United States showed that the implementation of suppression policies, which included lockdown, had a net benefit of about $298.70 billion compared to no intervention [79]. Six studies identified this strategy as a dominated alternative. In two articles, mitigation strategy [78] and paid sick leave for treatment [68] were introduced as dominant alternatives compared to this strategy. And in another article, lockdown under three alternative scenarios (income growth of 6%, 7% and 8%) was investigated and in all three scenarios the net benefit was less than zero [80]. In two studies, it was found that in implementing this strategy, the value of ICER was much higher than the threshold of willingness to pay [20, 81]. Another study evaluated the lockdown strategy in two conservative and generous scenarios and showed that the total cost per QALY was $32,489.78 and $18,049.23, respectively. In general, it was found that the cost of quarantine is 25 times higher than its benefit and none of the scenarios were cost-effective [82].

#### Social distancing strategy (S4)

The strategy of social distancing in combination with other interventions or individually was investigated in six articles. By comparing different forms of this strategy with other strategies or no intervention, it was found that although it was less cost-effective than vaccine [83], cleaning surfaces [69], and a combined mitigation strategy [78], this strategy was expressed as the dominant option in three studies in the United States.

A study with the CBA method showed that implementing social distancing compared to not implementing this intervention leads to $5.44 trillion in net monetary benefit [22]. Also, the results of a CEA study indicated that social distancing with masks was a more cost-effective solution than using masks alone or laboratory screening; this combined strategy saved $177.99 per infection averted and $51,512.4 per QALY, compared to these two alternatives [67]. Another study with CBA method showed that the combined strategy of limited reopening with social distancing was the superior option compared to the full reopening strategy to achieve herd immunity, and the ICER was $130,875 per QALY [84].

#### Personal protective equipment strategy (S5)

Different forms of personal protective equipment strategy (individually or in combination with other interventions) were investigated in seven articles. The single strategy of isolation and quarantine [76], the combined strategy of social distancing with masks [67], hand hygiene [85], and cleaning surfaces [69], were introduced as more cost-effective solutions than PPE. As a result of comparing the cost-effectiveness of personal protective equipment strategy with other strategies, or no intervention, or comparing its different forms together, this strategy was introduced as the dominant strategy in four studies.

In the study that was conducted with the CEA method, it was shown that adequate provision of personal protective equipment compared to inadequate provision of it in health workers, led to a saving of $61.01 per COVID-19 case prevented and $4,456.36 per life saved [86]. In another similar study with the same method in Kenya, it was shown that adequate supply compared to inadequate supply of PPE in health workers resulted in savings of $54.11 per case prevented and $393.66 per death prevented [87]. Also, a CBA study in New Zealand found that the implementation of the combined mitigation strategy, which included the use of PPE on public transportation, was more cost-effective than the lockdown strategy; with the implementation of the lockdown strategy, the cost per QALY was $960,405.60, which was much higher than the willingness-to-pay threshold [78]. A study from China, which was conducted with the CEA method, showed that the joint strategy of personal protection and isolation-and-quarantine was the superior option compared to the strategy of personal protection and community containment and no intervention. And this combined strategy saved $1,000 per case prevented [76].

#### Therapeutic intervention strategy (S6)

The strategy of therapeutic interventions in combination with other interventions or individually was investigated in 9 studies. Only one study found that the therapeutic intervention was less cost-effective than the competing option. In this study, the vaccine for COVID-19 was shown to be superior to the treatment for it [83]. In eight other studies, different forms of therapeutic intervention strategies were compared together, with no intervention, or with standard care.

Two of these studies were conducted in the United States using the CEA method. It was found that oxygen support with a ventilator was superior to no oxygen support, with an ICER of $8,508.70 per QALY [88]. It was also observed that the use of statin was a more cost-effective solution compared to not using it. Patients who received statin had lower costs ($33,109.28 vs. $34,779.24) and greater effectiveness (1.73 vs. 1.71). [89] In another study from this country, using the CUA method, it was shown that the choice of "Dexamethasone for all patients" was a superior option compared to its alternatives (Remdesivir to all patients, Remdesivir in only moderate and only severe infections, Remdesivir in moderate/Dexamethasone in severe infections, Dexamethasone in severe infections) [90]. Similarly, a study in Africa used the CEA method and reported an ICER of $241.64 per death prevented. The results of this study indicated that choosing Dexamethasone alone was more cost-effective than competing options for both non-ventilated and ventilated patients . Also, another study from the United Kingdom using the CEA method showed that if the patient meets the criteria for treatment with Dexamethasone, drug therapy with Dexamethasone is a more cost-effective option than not using it, and the ICER value is lower than the willingness-to-pay threshold per QALY ($27,768.56) [91]. A study from China with a similar methodology reported an ICER value of $2,239.86 per QALY and stated that the Remdesivir regimen was the dominant option compared to the standard of care [75]. A study from the Philippines, using the CBA method, found that using paid sick leave for treatment was superior to the two options of closing face-to-face schools and a combined TTQ strategy (benefit–cost ratio of 10 vs. 0.011 and 1.20, respectively) [68]. A study in Germany also using the CEA method, expressed the ICER value of $29,109.55 per life year gained, and stated that providing additional capacity by increasing ICU (Intensive Care Units) beds is more cost-effective than no intervention [92].

#### Vaccination strategy (S7)

Vaccination strategy has been studied in five articles in comparison with other strategies or no intervention. And in all these studies, various forms of this strategy have been described as cost-effective.

Two of the studies were conducted in the United States using the CEA method. In one of these, vaccination was found to be more cost-effective compared to no vaccination, with an ICER of $8,585.40 per QALY [93]. And in another study from this country, vaccination was presented as the superior option compared to no vaccination, therapeutic interventions, and social distancing (ICER8 = $837.60 per QALY) [83]. Similarly, a study in Turkey with the same methodology reported that the ICER was $686.20 per QALY and stated vaccination was a better option than no vaccination [11]. Also, a study from the United Kingdom, using the CBA method, showed that the net monetary value of vaccination compared to no vaccination ranged from $464.70 billion to $1.52 billion (in two scenarios, best-case scenario and worst-case scenario), and vaccination was introduced as a more cost-effective option [94]. In a study from China, using the CUA method, Moderna, Pfizer, AstraZeneca vaccines and no interventions were compared. The results of this study showed that the ICUR per QALY ($) was -321.14, -356.75, and -341.43, respectively [21].

#### Travel restriction strategy (S8)

The travel restriction strategy was compared with other strategies or no intervention in four studies. Although in one study the combined form of this strategy was less cost-effective than the joint strategy of personal protection and isolation and quarantine [76], it was expressed as the dominant strategy compared to the alternatives in three studies.

A study from China using the CUA method showed that the net monetary benefit (billion/$) for no-delayed implementation of movement restriction policies compared to one-week, two-week, and four-week delayed implementation was -394.04, -680.52, -941.15, and -3,397.46, respectively [95]. Another study from the United States, using the CBA method, stated that the implementation of state suppression policies, which include severe travel restrictions, resulted in a net benefit of $298.70 to $385.61 compared to no intervention [79]. Also, a study with the CBA method in New Zealand showed that the implementation of the mitigation strategy, which included transportation restrictions, was more cost-effective compared to the lockdown strategy. In implementing the lockdown strategy, the cost per QALY was $960,405.60, which was much higher than the willingness-to-pay threshold [78].

## Discussion

The focal point of this systematic review was the economic evaluation of strategies against COVID-19, and for this purpose, 36 articles were reviewed. In general, the solutions to deal with COVID-19 were placed in eight general categories (screening and diagnostic tests, quarantine and isolation, therapeutic interventions, social distancing, personal protective equipment, lockdowns, vaccination, and travel restriction). Screening and diagnostic tests, quarantine and isolation, therapeutic interventions were the most common strategies investigated in the studies.

In the comparison between different strategies against COVID-19, the evidence showed that the strategy of screening and diagnostic tests has a clear advantage over no intervention. One study compared this strategy with no intervention in the general population and found that the ICER per infection averted and per death averted were $8,260.83 and $1,497,210, respectively. It was found that even the use of high-frequency rapid home testing as an inexpensive and imperfect test can significantly help control the epidemic and be considered as part of the national containment strategy [23]. Similarly, a massive and rapid antigen testing program in Slovakia appears to have helped to a reduction in COVID-19 cases beyond what would have been expected through standard infection control measures [96]. In another study in Spain, it was shown that the use of a combined TTQ strategy in high-risk individuals is a preferable option compared to no intervention. In this study, it was found that in the long term, for each unit of cost spent on using this strategy, including and excluding the monetary benefit of health and morbidity, 19 and 7 times the benefit is obtained, respectively [73]. And the results of this study in Spain were closer to the values reported by Cutler and Summers, when monetary health gains were included [3].

Regarding the strategy of quarantine and isolation, it was found that using this strategy is a superior option compared to no intervention, especially when it is used in combination with the form of TTQ strategy [72, 73]. Evidence reveals that this combined strategy has been 19 times more profitable than its cost [73]. One study showed that the implementation of the isolation program after screening in the university community is essential and saves $8,271.30 per infection prevented [72]. In line with these results, a study in Colombia also stated that the use of Test-Trace-Isolate programs, compared to no intervention, reduced mortality by 67% and saved 1045 dollars per case from a social perspective [97]. Other findings showed that the use of quarantine and isolation strategy in the general population in certain conditions can be superior to the use of personal protective equipment. In sporadic and cluster outbreaks, isolation of infectious cases and quarantine of exposed people with infection was the most cost-effective measure (compared to PPE). However, when this strategy was used in combination with personal protective equipment, it was more cost-effective than using it alone. It was found that the cost-effectiveness of the quarantine and isolation strategy was very sensitive to the quarantine delay time, and when the quarantine delay time was more than 5 days, other alternatives (including personal protection and community containment) were preferable [76]. Similarly, other evidence has shown that the combined strategy of mask use with quarantine has been an effective strategy for controlling COVID-19 [67, 98].

Although the lockdown strategy as part of government repression policies, has been introduced as superior compared to non-intervention [79], it was stated as a non-cost-effective strategy in most studies [20, 80–82]. In a study, the cost of its implementation was estimated to be 25 times higher than its benefit [82]. And in another study, the net benefit of its implementation was stated to be less than zero [80]. Similar studies have shown that the implementation of a three-month lockdown in the UK is likely to result in a loss of 68 billion pounds to 547 billion pounds [99, 100]. It should be noted that none of these two studies considered the potential health consequences of the collapse of the health care system. Comparison between studies is challenging and should be done with caution, not only because of differences in context, but also because of methodological variation, such as the use of different health outcomes and variation in costs [101].

The results clearly showed that the implementation of social distancing is cost-effective compared to not implementing it and leads to $5.44 trillion in net monetary benefits [22]. Similar to these results, a study from Indonesia showed that social distancing could potentially save $415 billion to $699 billion and lead to a reduction in the total number of outpatients, non-ICU and ICU hospitalizations, and deaths due to COVID-19 [102]. Also, the results showed that the implementation of the social distancing strategy along with personal protective equipment (wearing a mask) was preferable to using personal protective equipment alone [67]. Similarly, in one study, it was noted that wearing a mask is a strong complement to social distancing; using computational fluid dynamics (CFD) technology, it was shown that social distancing can be reduced to 0.5 m if a mask is used [103].

Regarding the strategy of personal protective equipment, the results showed that an adequate supply of this equipment, especially for health workers, was very cost-effective compared to no intervention or, in other words, inadequate supply [86, 87]. Also, other results indicated that using this strategy in combination with other interventions such as wearing masks and travel restrictions for high-risk groups can be a better option than lockdown [78]. Evidence shows that the use of personal protective equipment can be cost-effective depending on the context. In a study that was conducted to investigate the level of protection of health workers in the delivery department, it was shown that for planned cesarean delivery, the implementation of universal PPE is a preferable option to screening [104]. Another study was conducted to determine the level of protection of health care workers in the endoscopy department and found that the cost-effectiveness of the PPE strategy decreased with an increasing prevalence rate [105].

In the review of studies related to therapeutic intervention strategy, it was found that often different forms of this strategy were compared with each other and not with other strategies. The results showed that the use of oxygen support with a ventilator [88], prescription of the statin [89], and Remdesivir regimen [75] were preferable options compared to routine care standards, provided that the patient had the necessary clinical conditions for prescription. Evidence has revealed that the price of Remdesivir is too high for its expected health gains, and if it leads to a reduction in mortality in patients with COVID-19, it may be considered a cost-effective intervention [106]. In addition, the results indicated that prescribing Dexamethasone for all patients (patients with low, moderate or severe infection and patients requiring ventilation or not requiring ventilation), provided that the indications for Dexamethasone therapy are followed, is probably a cost-effective option [90, 91]. Also, the results revealed that providing infrastructure to facilitate receiving medical services during the Corona pandemic was a cost-effective measure [68, 92]. A study found that the administration of Dexamethasone reduced mortality among people receiving invasive mechanical ventilation or oxygen alone [107]. In another study, it was mentioned that treatment with Dexamethasone in moderate-to-severe pneumonia reduces mortality, and since this drug is cheap and widely available, it can have a significant effect on patients with COVID-19 [108].

Reviewing studies on vaccination strategies, the results clearly revealed that vaccination is more cost-effective than no vaccination [11, 21, 93, 94]. Also, the results indicated the superiority of vaccination over therapeutic interventions, social distancing and no intervention [83]. Similarly, other evidence indicates the clinical efficacy and economic value of COVID-19 vaccination not only in high-income countries but also in middle- and low-income countries [109, 110]. A study showed that there was a strong interaction between social distancing and vaccination so that the right combination of these two effectively reduces hospitalization. In particular, prioritizing vaccines to the elderly (60 +) before adults (20–59) is more effective when social distancing is applied to adults or uniformly [111].

Regarding the travel restriction strategy, the results showed that its implementation as one of the measures of state suppression policies was cost-effective compared to no intervention [79]. Also, it was found that its delayed implementation greatly reduces its cost-effectiveness [95]. There was also evidence of its superiority over the lockdown strategy [78]. In line with these results, a study has shown that national and international travel restrictions in China have been effective in curbing the spread of COVID-19. And it was found that these restrictions are most effective when they are implemented early in the outbreak [112]. Similarly, in another study, it has been stated that due to the severe consequences of national lockdown, this intervention should be reduced and other non-pharmacological interventions should be used [113].

This research had limitations. The study results were limited to articles published in English, which indicates a potential limitation. In the different studies, the analysis method, cost type, time horizons and information sources were very different, so it was difficult to generalize the results to other settings.

## Conclusion

This systematic review aimed to summarize the economic evaluation evidence related to strategies against COVID-19. Based on the results of this systematic review, it seems that all strategies are likely to be more cost-effective against COVID-19 than no intervention. Vaccination was the most cost-effective, and decisions about lockdown strategy should be made with more caution, as there was conflicting evidence of its cost-effectiveness.

This useful evidence can potentially provide insight to policy-makers to decide on the introduction of optimal containment measures both in subsequent waves of the current epidemic and in handling possible future health crises.

## Data Availability

Not applicable.

## Abbreviations

GDP: Gross Domestic Product
SIR: Superiority and Inferiority Ranking
CHEERS: Consolidated Health Economic Evaluation Reporting Standards
CEA: Cost-Effectiveness Analysis
CUA: Cost Utility Analysis
CBA: Cost Benefit Analysis
DALYs: Disability Adjusted Life Years
YLDs: Years Lost due to Disability
YLLs: Years of Life Lost
QALY: Quality Adjusted Life Years
WHO: World Health Organization
U.S: United States
UK: United Kingdom
PICOS: Population–Intervention-Comparison–Outcomes- Study Design
MeSH: Medical Subject Headings
PRISMA: Preferred Reporting Items for Systematic reviews and Meta-Analyses
COVID-19: Coronavirus Disease 2019
IHME: Health Metrics and Evaluation
ICER: Incremental Cost-Effectiveness Ratio
NMB: Net Monetary Benefit
CBR: Benefit–Cost Ratio
RT-PCR: (Reverse Transcription Polymerase Chain Reaction)
TTQ: Test-Tracking-Quarantine
HCWs: Health Care Workers
PPE: Personal Protective Equipment
QALY: Quality Adjusted Life Years
NPIs: Non-Pharmacologic Interventions
PSA: Probabilistic Sensitivity Analyses
WTP: Willingness-To-Pay
WELLBYs: Wellbeing Years
Rs: Rupees
US: United States
UK: United Kingdom
ICU: Intensive Care Units
SEIR: Susceptible Exposed-Infected-Recovered
MRPs: Movement restriction policies
RMB: Renminbi
ICUR: Incremental Cost-Utility Ratio
SIR: Susceptible-Infected-Recovered
NSW: New South Wale
WA: Western Australia

## References

[1] Cucinotta D, Vanelli M (2020). WHO Declares COVID-19 a Pandemic. Acta Biomed.

[2] Nicola M, Alsafi Z, Sohrabi C, Kerwan A, Al-Jabir A, Iosifidis C (2020). The socio-economic implications of the coronavirus pandemic (COVID-19): a review. Int J Surg.

[3] Cutler DM, Summers LH (2020). The COVID-19 pandemic and the $16 trillion virus. JAMA.

[4] Inoue H, Todo Y (2020). The propagation of economic impacts through supply chains: the case of a mega-city lockdown to prevent the spread of COVID-19. PLoS One.

[5] Bartsch SM, Ferguson MC, McKinnell JA, O’shea KJ, Wedlock PT, Siegmund SS, et al. The potential health care costs and resource use associated with COVID-19 In the United States: a simulation estimate of the direct medical costs and health care resource use associated with COVID-19 infections in the United States. Health Aff. 2020;39(6):927–35. 10.1377/hlthaff.2020.00426 PMC11027994 32324428

[6] Forsythe S, Cohen J, Neumann P, Bertozzi SM, Kinghorn A (2020). The economic and public health imperatives around making potential coronavirus disease–2019 treatments available and affordable. Value Health.

[7] Jo M-W, Go D-S, Kim R, Lee SW, Ock M, Kim Y-E (2020). The burden of disease due to COVID-19 in Korea using disability-adjusted life years. J Korean Med Sci.

[8] Oh I-H, Ock M, Jang SY, Go D-S, Kim Y-E, Jung Y-S (2020). Years of life lost attributable to COVID-19 in high-incidence countries. J Korean Med Sci.

[9] Quast T, Andel R, Gregory S, Storch EA (2022). Years of life lost associated with COVID-19 deaths in the USA during the first 2 years of the pandemic. J Public Health (Oxf).

[10] Elekhnawy E, Kamar AA, Sonbol F (2021). Present and future treatment strategies for coronavirus disease 2019. Futur J Pharm Sci.

[11] Hagens A, İnkaya AÇ, Yildirak K, Sancar M, van der Schans J, AcarSancar A (2021). COVID-19 vaccination scenarios: a cost-effectiveness analysis for Turkey. Vaccines.

[12] AlFattani A, AlMeharish A, Nasim M, AlQahtani K, AlMudraa S (2021). Ten public health strategies to control the COVID-19 pandemic: the Saudi Experience. IJID Reg.

[13] Yan B, Zhang X, Wu L, Zhu H, Chen B (2020). Why do countries respond differently to COVID-19? A comparative study of Sweden, China, France, and Japan. Am Rev Public Adm.

[14] Porter C, Favara M, Sánchez A, Scott D (2021). The impact of COVID-19 lockdowns on physical domestic violence: Evidence from a list randomization experiment. SSM - Population Health.

[15] Khasawneh AI, Humeidan AA, Alsulaiman JW, Bloukh S, Ramadan M, Al-Shatanawi TN (2020). Medical students and COVID-19: knowledge, attitudes, and precautionary measures a descriptive study from Jordan. Front Public Health..

[16] Ogolodom MP, Mbaba AN, Alazigha N, Erondu O, Egbe N, Golden I, et al. Knowledge, attitudes and fears of healthcare workers towards the Corona virus disease (COVID-19) pandemic in South-South, Nigeria. Health Sci J. 2020;19(2):1-10.

[17] Henning AM, Thomas NJ, Williams DC, Shore DM, Memmi ME, Wang L, Effect of Coronavirus Disease,. (COVID-19), a nationwide mass casualty disaster on intensive care units: clinical outcomes and associated cost-of-care. Disaster Med Public Health Prep. 2019;2022:1–6. 10.1017/dmp.2022.159 PMC9353234 35703087

[18] Chen H, Shi L, Zhang Y, Wang X, Jiao J, Yang M (2021). Response to the COVID-19 pandemic: comparison of strategies in six countries. Front Public Health.

[19] Pandey KR, Subedee A, Khanal B, Koirala B (2021). COVID-19 control strategies and intervention effects in resource limited settings: a modeling study. PLoS ONE.

[20] Shlomai A, Leshno A, Sklan EH, Leshno M (2021). Modeling social distancing strategies to prevent SARS-CoV-2 spread in Israel: a cost-effectiveness analysis. Value in Health.

[21] Wang WC, Fann JC, Chang RE, Jeng YC, Hsu CY, Chen HH (2021). Economic evaluation for mass vaccination against COVID-19. J Formos Med Assoc.

[22] Thunström L, Newbold SC, Finnoff D, Ashworth M, Shogren JF (2020). The benefits and costs of using social distancing to flatten the curve for COVID-19. J Benefit Cost Analysis.

[23] Paltiel AD, Zheng A, Sax PE (2021). Clinical and economic effects of widespread rapid testing to decrease SARS-CoV-2 transmission. Ann Intern Med.

[24] Reddy KP, Shebl FM, Foote JH, Harling G, Scott JA, Panella C, et al. Cost-effectiveness of public health strategies for COVID-19 epidemic control in South Africa: a microsimulation modelling study. Lancet Global Health. 2021;9(2):e120–9. 10.1016/S2214-109X(20)30452-6 PMC7834260 33188729

[25] Persad G, Pandya A (2022). A Comprehensive COVID-19 Response—the need for economic evaluation. N Engl Med..

[26] Lessard C, Contandriopoulos A-P, Beaulieu M-D (2009). The role of economic evaluation in the decision-making process of family physicians: design and methods of a qualitative embedded multiple-case study. BMC Fam Pract.

[27] Flaxman S, Mishra S, Gandy A, Unwin HJT, Mellan TA, Coupland H (2020). Estimating the effects of non-pharmaceutical interventions on COVID-19 in Europe. Nature.

[28] Moher D, Liberati A, Tetzlaff J, Altman DG (2010). Preferred reporting items for systematic reviews and meta-analyses: the PRISMA statement. Int J Surg.

[29] Husereau D, Drummond M, Petrou S, Carswell C, Moher D, Greenberg D (2013). Consolidated health economic evaluation reporting standards (CHEERS) statement. BMC Med.

[30] Shlomai A, Leshno A, Sklan EH, Leshno MJM (2020). Cost-effectiveness analysis of social distancing strategies to prevent SARS-CoV2 spread.

[31] Zhao J, Jin H, Li X, Jia J, Zhang C, Zhao H (2021). Disease burden attributable to the first wave of COVID-19 in china and the effect of timing on the cost-effectiveness of movement restriction policies. Value Health.

[32] Hagens A, İnkaya AÇ, Yildirak K, Sancar M, van der Schans J, Acar Sancar A (2021). COVID-19 Vaccination Scenarios: A Cost-Effectiveness Analysis for Turkey. Vaccines (Basel).

[33] Wang Q, Shi N, Huang J, Yang L, Cui T, Ai J (2022). Cost-Effectiveness of public health measures to control COVID-19 in China: a microsimulation modeling study. Front Public Health.

[34] Wang WC, Fann JC, Chang RE, Jeng YC, Hsu CY, Chen HH (2021). Economic evaluation for mass vaccination against COVID-19. J Formos Med Assoc.

[35] Padula WV, Malaviya S, Reid NM, Cohen BG, Chingcuanco F, Ballreich J (2021). Economic value of vaccines to address the COVID-19 pandemic: a US cost-effectiveness and budget impact analysis. J Med Econ.

[36] Kohli M, Maschio M, Becker D, Weinstein MC. The potential public health and economic value of a hypothetical COVID-19 vaccine in the United States: Use of cost-effectiveness modeling to inform vaccination prioritization. 2021;39(7):1157–64. 10.1016/j.vaccine.2020.12.078 PMC7832653 33483216

[37] Jiang Y, Cai D, Chen D, Jiang S. The cost-effectiveness of conducting three versus two reverse transcription-polymerase chain reaction tests for diagnosing and discharging people with COVID-19: evidence from the epidemic in Wuhan, China. BMJ Glob Health. 2020;5(7). 10.1136/bmjgh-2020-002690 PMC7385750 32694221

[38] Melia A, Lee D, Mahmoudi N, Li Y, Paolucci FJJoR, Management F. Cost-Effectiveness Analysis of COVID-19 Case Quarantine Strategies in Two Australian States: New South Wales and Western Australia. J Risk Financ Manage. 2021;14(7):305.

[39] Risko N, Werner K, Offorjebe OA, Vecino-Ortiz AI, Wallis LA, Razzak JJPO (2020). Cost-effectiveness and return on investment of protecting health workers in low-and middle-income countries during the COVID-19 pandemic. PLoS One..

[40] Thunström L, Newbold SC, Finnoff D, Ashworth M, Shogren JFJJoB-CA. The benefits and costs of using social distancing to flatten the curve for COVID-19. Trans R Soc Trop Med Hyg. 2020;11(2):179–95.

[41] González López-Valcárcel B, Vallejo-Torres L (2021). The costs of COVID-19 and the cost-effectiveness of testing. Appl Econ Analysis.

[42] Losina E, Leifer V, Millham L, Panella C, Hyle EP, Mohareb AM, et al. College campuses and COVID-19 mitigation: clinical and economic value. medRxiv [Preprint]. 2021;174(4):472–83. 10.7326/M20-6558 PMC7755069 33347322

[43] Bagepally BS, Haridoss M, Natarajan M, Jeyashree K, Ponnaiah MJCe, health g. Cost-effectiveness of surgical mask, N-95 respirator, hand-hygiene and surgical mask with hand hygiene in the prevention of COVID-19: Cost effectiveness analysis from Indian context. Clin Epidemiol Glob Health. 2021;10:100702. 10.1016/j.cegh.2021.100702 PMC7859732 33558852

[44] Sheinson D, Dang J, Shah A, Meng Y, Elsea D, Kowal S (2021). A Cost-Effectiveness Framework for COVID-19 Treatments for Hospitalized Patients in the United States. Adv Ther.

[45] Paltiel AD, Zheng A, Sax PE (2021). Clinical and Economic Effects of Widespread Rapid Testing to Decrease SARS-CoV-2 Transmission. Ann Intern Med.

[46] Abdalhamid B, Bilder CR, Garrett JL, Iwen PC (2020). Cost effectiveness of sample pooling to test for SARS-CoV-2. J Infect Dev Ctries..

[47] Neilan AM, Losina E, Bangs AC, Flanagan C, Panella C, Eskibozkurt GE (2020). Clinical impact, costs, and cost-effectiveness of expanded SARS-CoV-2 testing in Massachusetts.

[48] Asamoah JKK, Owusu MA, Jin Z, Oduro F, Abidemi A, Gyasi EOJC, Solitons, et al. Global stability and cost-effectiveness analysis of COVID-19 considering the impact of the environment: using data from Ghana. Chaos Solitons Fractals. 2020;140:110103. 10.1016/j.chaos.2020.110103 PMC7351453 32834629

[49] Ryan AJAaS. A Cost–Benefit Analysis of the COVID-19 Lockdown in Ireland. 2021.

[50] Águas R, Mahdi A, Shretta R, Horby P, Landray M, White LJJm. The potential health and economic impact of dexamethasone treatment for patients with COVID-19. 2020.

[51] Rowthorn R, Maciejowski JJORoEP. A cost–benefit analysis of the COVID-19 disease. Oxford Rev Econ Policy. 2020;36(Supplement_1):S38-S55. 10.1093/oxrep/graa030 PMC7499782 40504226

[52] Paltiel AD, Zheng A, Walensky RPJm. COVID-19 screening strategies that permit the safe re-opening of college campuses. 2020. 10.1001/jamanetworkopen.2020.16818 PMC7395236 32735339

[53] Broughel J, Kotrous MJPo. The benefits of coronavirus suppression: A cost-benefit analysis of the response to the first wave of COVID-19 in the United States. 2021;16(6):e0252729. 10.1371/journal.pone.0252729 PMC8174714 34081757

[54] Gandjour AJM (2020). How much reserve capacity is justifiable for hospital pandemic preparedness? A cost-effectiveness analysis for COVID-19 in Germany.

[55] Schonberger RB, Listokin YJ, Ayres I, Yaesoubi R, Shelley ZRJM (2020). Cost benefit analysis of limited reopening relative to a herd immunity strategy or shelter in place for SARS-CoV-2 in the United States.

[56] Sandmann FG, Davies NG, Vassall A, Edmunds WJ, Jit M, Sun FY (2021). The potential health and economic value of SARS-CoV-2 vaccination alongside physical distancing in the UK: a transmission model-based future scenario analysis and economic evaluation. Lancet Infect Dis.

[57] Dutta M, Husain ZJM (2020). What cost decisiveness? A cost benefit analysis of the lockdown to contain COVID-19 in India.

[58] Kazungu J, Munge K, Werner K, Risko N, Ortiz AV, Were V (2021). Examining the Cost-effectiveness of Personal Protective Equipment for Formal Healthcare Workers in Kenya during the COVID-19 Pandemic. BMC Health Serv Res.

[59] Chow R, Prsic EH, Shin HJJm. Cost-Effectiveness Analysis of Statins for Treatment of Hospitalized COVID-19 Patients. 2021. 10.21037/apm-21-2797 35400155

[60] Jo Y, Jamieson L, Edoka I, Long L, Silal S, Pulliam JRC, Moultrie H, Sanne I, Meyer-Rath G, Nichols BE. Cost-effectiveness of Remdesivir and Dexamethasone for COVID-19 Treatment in South Africa. Open Forum Infect Dis. 2021;29;8(3). 10.1093/ofid/ofab040 PMC7928624 33732750

[61] Jiang Y, Cai D, Chen D, Jiang S, Si L (2021). Wu J Economic evaluation of Remdesivir for the treatment of severe COVID-19 patients in China under different scenarios. Br J Clin Pharmacol.

[62] Congly SE, Varughese RA, Brown CE, Clement FM, Saxinger LJSR. Treatment of moderate to severe respiratory COVID-19: a cost-utility analysis. Sci Rep. 2021;11(1):1–7. 10.1038/s41598-021-97259-7 PMC8423816 34493774

[63] Lally M (2021). The costs and benefits of COVID-19 lockdowns in New Zealand. MedRxiv..

[64] Raitzer D, Lavado RF, Rabajante J (2020). Cost–Benefit Analysis of Face-to-Face Closure of Schools to Control COVID-19 in the Philippines.

[65] Seguí FL, Cuxart OE, i Villar OM, Guillamet GH, Gil N, Bonet JM (2021). Cost-Benefit Analysis of the COVID-19 Asymptomatic Mass Testing Strategy in the North Metropolitan Area of Barcelona.

[66] Jafari A, Rezapour A, Hajahmadi M (2018). Cost-effectiveness of B-type natriuretic peptide-guided care in patients with heart failure: a systematic review. Heart Fail Rev.

[67] Losina  E, Leifer V, Millham L, Panella C, Hyle EP, Mohareb AM (2021). College campuses and COVID-19 mitigation: clinical and economic value. Ann Intern Med.

[68] Raitzer D, Lavado RF, Rabajante J (2020). Cost–benefit analysis of face-to-face closure of schools to control COVID-19 in the Philippines.

[69] Asamoah JKK, Owusu MA, Jin Z, Oduro F, Abidemi A, Gyasi EO (2020). Global stability and cost-effectiveness analysis of COVID-19 considering the impact of the environment: using data from Ghana. Chaos, Solitons Fractals.

[70] Abdalhamid B, Bilder CR, Garrett JL, Iwen PC (2020). Cost effectiveness of sample pooling to test for SARS-CoV-2. J Infect Dev Ctries.

[71] Neilan AM, Losina E, Bangs AC, Flanagan C, Panella C, Eskibozkurt G, et al. Clinical impact, costs, and cost-effectiveness of expanded severe acute respiratory syndrome Coronavirus 2 testing in Massachusetts. Clin Infect Dis. 2020;73(9):e2908–17. 10.1093/cid/ciaa1418 PMC7543346 32945845

[72] Paltiel AD, Zheng A, Walensky RP. COVID-19 screening strategies that permit the safe re-opening of college campuses. medRxiv. 2020. Preprint. 10.1001/jamanetworkopen.2020.16818 PMC7395236 32735339

[73] López-Valcárcel BG, Vallejo-Torres L. The costs of COVID-19 and the cost-effectiveness of testing. Appl Econ Analysis. 2021. Ahead-of-print.

[74] López Seguí F, Estrada Cuxart O, Mitjà i Villar O, Hernández Guillamet G, Prat Gil N, Maria Bonet J, et al. A cost-benefit analysis of the COVID-19 asymptomatic mass testing strategy in the north Metropolitan area of Barcelona. Int J Environ Res Public Health. 2021;18(13):7028. 10.3390/ijerph18137028 PMC8297108 34209328

[75] Jiang Y, Cai D, Chen D, Jiang S (2020). The cost-effectiveness of conducting three versus two reverse transcription-polymerase chain reaction tests for diagnosing and discharging people with COVID-19: evidence from the epidemic in Wuhan, China. BMJ Glob Health.

[76] Wang Q, Shi N, Huang J, Yang L, Cui T, Ai J (2022). Cost-Effectiveness of Public Health Measures to Control COVID-19 in China: A Microsimulation Modeling Study. Front Public Health.

[77] Melia A, Lee D, Mahmoudi N, Li Y, Paolucci F (2021). Cost-effectiveness Analysis of COVID-19 case quarantine strategies in two Australian States: New South Wales and Western Australia. J Risk Financ Manage.

[78] Lally M. The costs and benefits of COVID-19 lockdowns in New Zealand. medRxiv [Preprint]. 2021.

[79] Broughel J, Kotrous M (2021). The benefits of coronavirus suppression: a cost-benefit analysis of the response to the first wave of COVID-19 in the United States. PLoS ONE.

[80] Dutta M, Husain Z. What cost decisiveness? A cost benefit analysis of the lockdown to contain COVID-19 in India. medRxiv. 2020:2020.07.07.20148338.

[81] Rowthorn R, Maciejowski J. A cost–benefit analysis of the COVID-19 disease: Oxford Review of Economic Policy. 2020 Aug 29:graa030 10.1093/oxrep/graa030. 10.1093/oxrep/graa030 PMC7499782 40504226

[82] Ryan A (2021). A Cost–Benefit Analysis of the COVID-19 Lockdown in Ireland.

[83] Padula WV, Malaviya S, Reid NM, Cohen BG, Chingcuanco F, Ballreich J (2021). Economic value of vaccines to address the COVID-19 pandemic: a US cost-effectiveness and budget impact analysis. J Med Econ.

[84] Schonberger RB, Listokin YJ, Ayres I, Yaesoubi R, Shelley ZR. Cost benefit analysis of limited reopening relative to a herd immunity strategy or shelter in place for SARS-CoV-2 in the United States. MedRxiv. 2020. Preprint.

[85] Bagepally BS, Haridoss M, Natarajan M, Jeyashree K, Ponnaiah M (2021). Cost-effectiveness of surgical mask, N-95 respirator, hand-hygiene and surgical mask with hand hygiene in the prevention of COVID-19: Cost effectiveness analysis from Indian context. Clin Epidemiol Global Health.

[86] Risko N, Werner K, Offorjebe OA, Vecino-Ortiz AI, Wallis LA, Razzak J (2020). Cost-effectiveness and return on investment of protecting health workers in low-and middle-income countries during the COVID-19 pandemic. PLoS ONE.

[87] Kazungu J, Munge K, Werner K, Risko N, Vecino-Ortiz AI, Were V (2021). Examining the cost-effectiveness of personal protective equipment for formal healthcare workers in Kenya during the COVID-19 pandemic. BMC Health Serv Res.

[88] Sheinson D, Dang J, Shah A, Meng Y, Elsea D, Kowal S (2021). A cost-effectiveness framework for COVID-19 treatments for hospitalized patients in the United States. Adv Ther.

[89] Chow R, Prsic EH, Shin HJ. Cost-Effectiveness Analysis of Statins for Treatment of Hospitalized COVID-19 Patients. medRxiv. 2021;11(7):2285–90. 10.21037/apm-21-2797 35400155

[90] Congly SE, Varughese RA, Brown CE, Clement FM, Saxinger L (2021). Treatment of moderate to severe respiratory COVID-19: a cost-utility analysis. Sci Rep.

[91] Águas R, Mahdi A, Shretta R, Horby P, Landray M, White L (2021). Potential health and economic impacts of Dexamethasone treatment for patients with COVID-19. Nat Commun.

[92] Gandjour A. How much reserve capacity is justifiable for hospital pandemic preparedness? A cost-effectiveness analysis for COVID-19 in Germany. medRxiv. 2020. Preprint. 10.1007/s40258-020-00632-2 PMC7801567 33433853

[93] Kohli M, Maschio M, Becker D, Weinstein MC (2021). The potential public health and economic value of a hypothetical COVID-19 vaccine in the United States: Use of cost-effectiveness modeling to inform vaccination prioritization. Vaccine.

[94] Sandmann FG, Davies NG, Vassall A, Edmunds WJ, Jit M, Sun FY (2021). The potential health and economic value of SARS-CoV-2 vaccination alongside physical distancing in the UK: a transmission model-based future scenario analysis and economic evaluation. Lancet Infect Dis.

[95] Zhao J, Jin H, Li X, Jia J, Zhang C, Zhao H (2021). Disease burden attributable to the first wave of COVID-19 in China and the effect of timing on the cost-effectiveness of movement restriction policies. Value in Health.

[96] Pavelka M, Van-Zandvoort K, Abbott S, Sherratt K, Majdan M, group CC-w, et al. The effectiveness of population-wide, rapid antigen test based screening in reducing SARS-CoV-2 infection prevalence in Slovakia. MedRXiv. 2020:2020.12. 02.20240648.

[97] Ruiz YG, Vecino-Ortiz AI, Guzman-Tordecilla N, Peñaloza-Quintero RE, Fernández-Niño JA, Rojas-Botero M (2022). Cost-Effectiveness of the COVID-19 test, trace and isolate program in Colombia. Lancet Reg Health-Am.

[98] Vandepitte S, Alleman T, Nopens I, Baetens J, Coenen S, De Smedt D (2021). Cost-effectiveness of COVID-19 policy measures: a systematic review. Value in Health.

[99] Miles DK, Stedman M, Heald AH (2021). “Stay at Home, Protect the National Health Service, Save Lives”: a cost benefit analysis of the lockdown in the United Kingdom. Int J Clin Pract.

[100] Miles D, Stedman M, Heald A (2020). Living with COVID-19: balancing costs against benefits in the face of the virus. Natl Inst Econ Rev.

[101] Vandepitte S, Alleman T, Nopens I, Baetens J, Coenen S, De Smedt D (2021). Cost-Effectiveness of COVID-19 Policy Measures: A Systematic Review. Value Health.

[102] Suwantika AA, Dhamanti I, Suharto Y, Purba FD, Abdulah R (2022). The cost-effectiveness of social distancing measures for mitigating the COVID-19 pandemic in a highly-populated country: a case study in Indonesia. Travel Med Infect Dis.

[103] Deng Z, Chen Q (2022). What is suitable social distancing for people wearing face masks during the COVID-19 pandemic?. Indoor Air.

[104] Savitsky LM, Albright CM (2020). Preventing COVID-19 transmission on labor and delivery: a decision analysis. Am J Perinatol.

[105] Ebigbo A, Römmele C, Bartenschlager C, Temizel S, Kling E, Brunner J (2021). Cost-effectiveness analysis of SARS-CoV-2 infection prevention strategies including pre-endoscopic virus testing and use of high risk personal protective equipment. Endoscopy.

[106] Whittington MD, Pearson SD, Rind DM, Campbell JD (2022). The Cost-Effectiveness of Remdesivir for Hospitalized Patients With COVID-19. Value in Health.

[107] Group RC (2021). Dexamethasone in hospitalized patients with COVID-19. N Engl J Med.

[108] Villar J, Añón JM, Ferrando C, Aguilar G, Muñoz T, Ferreres J (2020). Efficacy of Dexamethasone treatment for patients with the acute respiratory distress syndrome caused by COVID-19: study protocol for a randomized controlled superiority trial. Trials.

[109] Utami AM, Rendrayani F, Khoiry QA, Alfiani F, Kusuma AS, Suwantika AA. Cost-Effectiveness Analysis of COVID-19 Vaccination in Low-and Middle-Income Countries. J Multidiscip Healthc. 2022;15:2067–76. 10.2147/JMDH.S372000 PMC9482370 36124175

[110] Li R, Liu H, Fairley CK, Zou Z, Xie L, Li X (2022). Cost-effectiveness analysis of BNT162b2 COVID-19 booster vaccination in the United States. Int J Infect Dis.

[111] Guerstein S, Romeo-Aznar V, Dekel Ma, Miron O, Davidovitch N, Puzis R (2021). The interplay between vaccination and social distancing strategies affects COVID19 population-level outcomes. PLoS Comput Biol.

[112] Grépin KA, Ho T-L, Liu Z, Marion S, Piper J, Worsnop CZ (2021). Evidence of the effectiveness of travel-related measures during the early phase of the COVID-19 pandemic: a rapid systematic review. BMJ Glob Health.

[113] Cascini F, Failla G, Gobbi C, Pallini E, Hui J, Luxi W (2022). A cross-country comparison of COVID-19 containment measures and their effects on the epidemic curves. BMC Public Health.

